# Biomechanical superiority of partial-thickness tendon split and bridging repair after BPTB graft harvest for ACL reconstruction

**DOI:** 10.1016/j.asmart.2026.04.001

**Published:** 2026-05-14

**Authors:** Nadhaporn Saengpetch, Khemchat Sawangworachat, Panya Aroonjarattham, Pinkawas Kongmalai

**Affiliations:** a Department of Orthopaedics, Faculty of Medicine, Ramathibodi Hospital, Mahidol University, Bangkok, 10400, Thailand; b Department of Mechanical Engineering, Faculty of Engineering, Mahidol University, Nakornpathom, 73170, Thailand; c Faculty of Medicine, Kasetsart University, 50, Ngamwongwan Road, Lat Yao, Chatuchak, Bangkok, 10900, Thailand

**Keywords:** ACL reconstruction, Biomechanical testing, Bone-patellar tendon-bone autograft, Partial-thickness tendon repair, Patellar tendon repair

## Abstract

**Purpose:**

Reconstruction of the anterior cruciate ligament (ACL) with a bone–patellar tendon–bone (BPTB) autograft leaves a central tendon defect, and whether this should be repaired remains debated. Conventional suturing techniques often show limited biomechanical benefit. This study evaluated the biomechanical efficacy of a novel Partial-Thickness Split and Bridging Repair (PTSBR) technique compared with non-repair in a cadaveric model.

**Methods:**

Twelve knees from six fresh-frozen human cadavers were randomized to repair or non-repair groups. In the repair group, PTSBR involved a longitudinal partial-thickness incision with superficial tendon bridging and interrupted vertical mattress sutures. All specimens were mounted and tested using an Instron universal testing machine. Primary outcomes included maximal load, maximal stress, stiffness, Young's modulus, tendon dimensions, and failure location. Statistical comparisons were performed using paired analyses with significance set at p < 0.05.

**Results:**

PTSBR demonstrated significantly higher Young's modulus [90.19 MPa (24.02–202.47) vs. 50.83 MPa (19.96–115.29), p = 0.028] and maximal stress [19.35 MPa (5.21–35.54) vs. 13.30 MPa (5.21–24.23), p = 0.046] compared with non-repair. No difference was observed in maximal load (p = 0.674) or stiffness. Tendon thickness was significantly reduced in the repair group (2.34 ± 0.20 mm vs. 2.69 ± 0.32 mm, p = 0.023). Failure occurred mainly at the tibial or patellar attachment, with no mid-substance ruptures.

**Conclusion:**

The PTSBR technique enhanced tensile stiffness and stress resistance compared with non-repair, likely due to improved tendon fiber alignment and load-sharing. These biomechanical advantages suggest that PTSBR may represent a clinically relevant advancement in managing patellar tendon defects after BPTB graft harvest.

## 1 Introduction

Reconstruction of the anterior cruciate ligament (ACL) using a bone-patellar tendon-bone (BPTB) autograft is a widely performed surgical technique due to its excellent graft strength and reliable bone-to-bone fixation.^1^, ^2^, ^3^ However, the procedure leaves a defect in the central third of the patellar tendon, raising debate about whether this defect should be repaired. Studies advocating for repair suggest that it could reduce the risk of patellar tendon rupture and extensor lag by improving tendon strength.^4^^,^^5^ Moreover, because quadriceps strength depends significantly on the width of the patellar tendon, with narrower widths being associated with greater quadriceps weakness, repairing the patellar tendon has been recommended in such cases.^6^^,^^7^

Nevertheless, multiple studies have demonstrated that repairing the defect does not significantly improve biomechanical properties^8^, ^9^, ^10^ or clinical outcomes.^11^^,^^12^ Radiographic studies indicate that the tendon gap gradually fills with tendon-like tissue, achieving near-complete healing within 2 to 3 years, albeit with some degree of scar tissue formation.^13^, ^14^, ^15^, ^16^ Systematic reviews further confirm that leaving the defect unrepaired is a viable and effective approach.^17^

Previous studies have primarily assessed repair techniques using conventional suturing methods, which typically involve directly approximating the edges of the tendon defect.^8^^,^^9^^,^^18^ While effective in closing the defect, these methods may face challenges such as suboptimal tendon alignment and limited load-sharing, which could contribute to variability in biomechanical outcomes.

To overcome these limitations, we developed a Partial-Thickness Split and Bridging Repair (PTSBR) technique. This approach is conceptually based on the principles of force-line optimization and load distribution in tendon biomechanics. By creating a controlled longitudinal partial-thickness incision in the residual tendon, PTSBR enables the superficial layers to be realigned and bridged across the central defect. Interrupted strong sutures placed at regular intervals then act to share load more effectively along the fiber direction, rather than simply closing the gap.

The purpose of this study was to evaluate the biomechanical efficacy of the PTSBR technique compared with non-repair in a controlled cadaveric model. We specifically examined whether PTSBR improves tensile properties such as stiffness and Young's modulus while preserving the integrity of the tendon mid-substance. We hypothesized that PTSBR would provide superior load-sharing and enhanced structural adaptation compared with leaving the defect unrepaired.

## 2 Methods

### 2.1 Study design and specimens

This biomechanical study was conducted using six fresh-frozen human cadavers, providing a total of 12 knees, in a controlled laboratory setting. Ethical approval was obtained from the institutional review board (Protocol Number: ID 06-59-56). Inclusion criteria required specimens to exhibit intact patellar tendons, no evidence of deformities, no prior knee surgery, no history of fractures, and no observable quadriceps muscle atrophy or imbalance. Exclusion criteria encompassed specimens with prior patellar tendon rupture, significant scarring or fibrosis in the patellar or tibial regions, previous knee surgical procedures, implanted hardware, severe osteoarthritic changes, ligamentous injuries, or signs of infection or tissue degradation. These criteria ensured homogeneity of specimens and mitigated confounding factors that could influence the biomechanical results.

### 2.2 Graft harvesting and repair technique

All surgical procedures were performed by a single senior fellow-trained knee surgeon to ensure consistency and precision. Cadaveric knee specimens were prepared using a midline incision. The middle third of the patellar tendon, along with patellar and tibial bone blocks, was harvested. Patellar bone blocks measured 10 mm in width and 25 mm in length, while tibial bone blocks were 10 mm in width and 35 mm in length. Following graft harvesting, each specimen was randomized to either the repair group, which utilized the Partial Thickness Tendon Split and Bridging Repair (PTSBR) technique, or the non-repair group. This paired design ensured balanced treatment allocation within each cadaver, allowing for direct biomechanical comparisons.

**Fig. 1:**
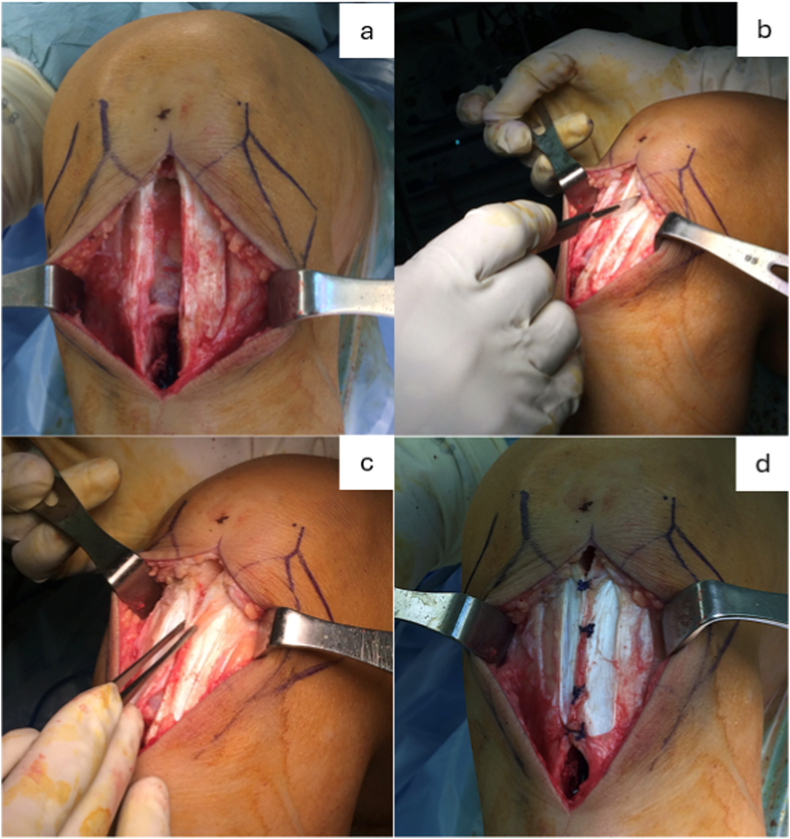
Intraoperative demonstration of the Partial-Thickness Split and Bridging Repair (PTSBR) technique. (a) Central patellar tendon defect after BPTB graft harvest. (b–c) Creation of longitudinal partial-thickness splits and mobilization of superficial tendon layers. (d) Final construct with interrupted vertical mattress sutures bridging the defect.

**Fig. 2:**
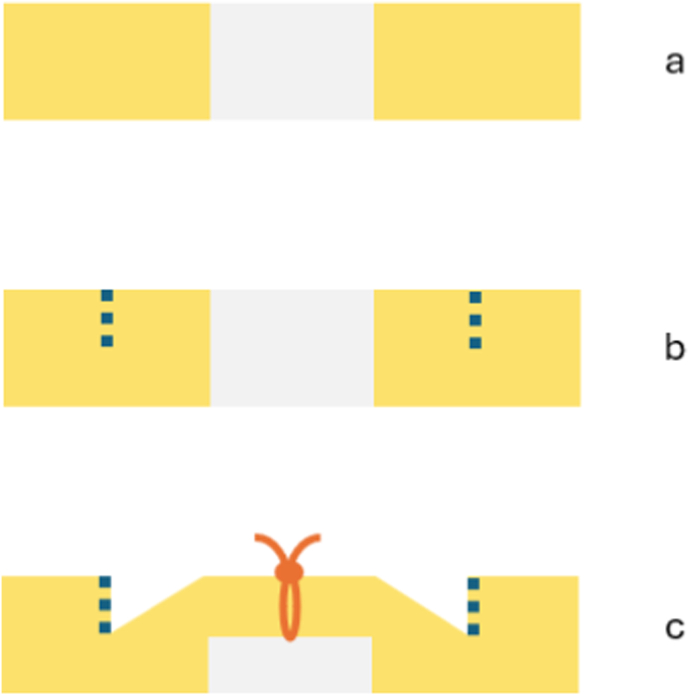
**Schematic illustration of the Partial-Thickness Split and Bridging Repair (PTSBR) technique.** (a) Cross-sectional view of the patellar tendon demonstrating the central defect created after BPTB graft harvest. (b) Longitudinal partial-thickness incisions extending along both sides of the remaining tendon to mobilize the superficial layers. (c) Approximation and bridging of the superficial tendon layers across the central defect using interrupted vertical mattress sutures.

In the repair group, the PTSBR technique involved a longitudinal partial-thickness incision corresponding to 50% of tendon thickness, measured with a digital caliper, along the length of the remaining tendon, extending from the proximal to distal ends on both sides of the patellar tendon (Fig. 1a–c). The superficial portions of the tendon were then approximated and bridged across the central defect using interrupted vertical mattress sutures with No. 2 Ethibond sutures (Ethicon US, LLC, USA), as demonstrated intraoperatively (Fig. 1d) and illustrated schematically in Fig. 2. Each suture was secured with a 4-throw square knot, and sutures were spaced at 10 mm intervals. The repair started at the lower pole of the patella and extended distally. In the non-repair group, the defect was left untreated following conventional practice.

### 2.3 Mounting and biomechanical testing preparation

**Fig. 3:**
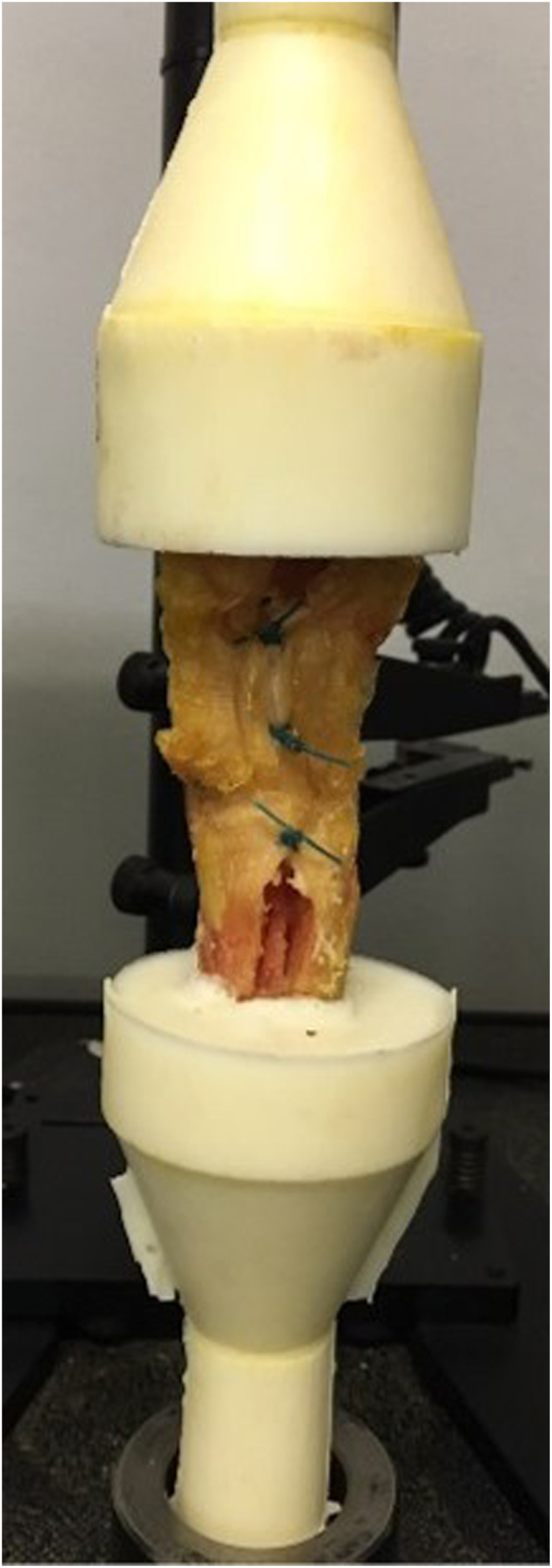
The PTSBR construct during biomechanical testing. The image demonstrates the patellar tendon following bone–patellar tendon–bone graft harvest and subsequent Partial-Thickness Split and Bridging Repair (PTSBR). The longitudinal partial-thickness split and interrupted vertical mattress sutures bridging the central defect are clearly visible. The specimen is mounted within the testing apparatus for axial load-to-failure evaluation.

The mounting material consisted of Epoxy resin F35 (Olin Epoxy, Houston, TX, USA), which was poured into a cone-shaped container around the bony parts of each specimen. The upper half of the patellar bone and a 5 cm length of the distal tibia were embedded in the epoxy resin. After sufficient consolidation, the prepared specimens were securely mounted to the tensioning arms of the Instron testing machine (Fig. 3). Tendon cross-sectional area was measured at the mid-substance using a digital caliper (accuracy 0.01 mm).

### 2.4 Biomechanical testing protocol

Biomechanical testing was conducted using the Instron 4467 universal testing machine (Illinois Toolworks Inc, Norwood, MA, USA). The setup ensured that the tendon was aligned anatomically for axial loading. Before testing, each specimen underwent preconditioning with a preload of 10 N for 10 cycles at a displacement rate of 10 mm/min to minimize viscoelastic effects. Tensile load-to-failure tests were conducted to assess the biomechanical performance of repaired and unrepaired tendons.

The primary parameters measured included maximal load, maximal stress, stiffness, Young's modulus, and failure location. Maximal load was defined as the highest force the tendon could sustain before failure, while maximal stress represented the force normalized by the tendon cross-sectional area, indicating its resistance to deformation. Stiffness was derived from the slope of the load-displacement curve within the elastic deformation range, reflecting the tendon's structural rigidity. Young's modulus was recorded to quantify the elastic properties and tensile stiffness of the tendon material. Additionally, the location of failure was carefully documented to identify whether it occurred at the repair site, midsubstance of the tendon, or the bone-tendon interface.

### 2.5 Statistical analysis

A paired *t*-test was used to calculate the required sample size based on previous data involving patellar tendons. Specifically, the study by Atkinson et al. reported mean ± standard deviation (SD) values for the non-augmented group as 0.51 ± 0.29 mm and for the augmented group as 0.39 ± 0.15 mm. The outcome variable under consideration is the displacement necessary to recruit 50% of tendon fibers, as this reflects critical tendon properties under tensile loading.^10^ To achieve sufficient statistical power, the Type I error (α) was set at 0.05 and the Type II error (β) at 0.3. The sample size was calculated using the formula: n = ((Z_(α/2) + Z_β)ˆ2 ∗σˆ2)/Δˆ2, where Δ represents the minimum detectable difference between the two groups, σ is the standard deviation of the differences, and Z represents the critical values corresponding to the chosen α and β levels. Based on this calculation, a total of 12 knees (6 paired specimens) was determined to be necessary to adequately power the study. This sample size ensures sufficient precision to detect meaningful differences between the groups while maintaining the predefined error thresholds.

Statistical analyses were performed using Stata software version 14.1 (StataCorp LLC, Texas, USA). Normality of data distribution was assessed using the Shapiro–Wilk test. Paired comparisons were conducted using the paired *t*-test for normally distributed data or the Wilcoxon signed-rank test for non-parametric data. The Kruskal-Wallis test was used to compare ranks across multiple groups. A p-value of <0.05 was considered statistically significant.

## 3 Results

Six cadaveric specimens, including four males and two females with a mean age of 67.83 years (range: 56-84 years), were analyzed. Biomechanical testing revealed that the repair group utilizing the PTSBR technique exhibited enhanced biomechanical properties compared to the non-repair group, including higher tensile stiffness, increased maximal stress, and reduced tendon thickness.

According to the Shapiro–Wilk test, tendon length, width, and thickness were normally distributed and are reported as mean ± standard deviation (SD), whereas Young's modulus, maximal load, maximal stress, and stiffness were non-normally distributed and are presented as median (range).

The ultimate tensile strength (maximal load) did not differ significantly between the two groups [repair group: 927.63 N (380.93–1763.72), non-repair group: 911.92 N (380.93–2013.37), p = 0.674]. However, the repair group demonstrated a significantly higher Young's modulus [repair group: 90.19 MPa (24.02–202.47), non-repair group: 50.83 MPa (19.96–115.29), p = 0.028], indicating improved tensile stiffness of the tendon material. Additionally, maximal stress was significantly higher in the repair group [repair group: 19.35 MPa (5.21–35.54), non-repair group: 13.30 MPa (5.21–24.23), p = 0.046].

Failure location analysis indicated differing patterns between groups. In the non-repair group, failure predominantly occurred at the tibial attachment (four cases) and less frequently at the patellar attachment (two cases). Conversely, in the repair group, failure was evenly distributed between the tibial (three cases) and patellar (three cases) attachments. Importantly, no mid-substance tears were observed in either group, indicating that the central tendon structure remained intact regardless of the repair method.

The thickness of the patellar tendon was significantly reduced in the repair group [repair group: 2.34 ± 0.20 mm, non-repair group: 2.69 ± 0.32 mm, p = 0.023]. There were no significant differences in tendon length, width, or stiffness between the groups. These findings are detailed in Table 1.

**Table 1:** Comparison of Biomechanical Parameters Between Repair and Non-Repair Groups Biomechanical parameters are presented as mean ± standard deviation (SD) for normally distributed variables and as median (range) for non-normally distributed variables, as determined by the Shapiro–Wilk test. Parameters include tendon length, width, thickness, Young's modulus, maximal load, maximal stress, and stiffness. Significant differences (p < 0.05) are denoted with an asterisk (∗). The repair group demonstrated significantly improved tendon thickness, Young's modulus, and maximal stress compared with the non-repair group.

Factors	Repair	Non-repair	P-value
Length (mm)	32.04 ± 5.63	32.28 ± 2.64	0.995
Width (mm)	22.10 ± 5.15	27.24 ± 9.70	0.336
Thickness (mm)	2.34 ± 0.20	2.69 ± 0.32	0.023∗
Young's modulus (MPa)	90.19 (24.02-202.47)	50.83 (19.96-115.29)	0.028∗
Maximal load (N)	927.63 (380.93-1763.72)	911.92 (380.93-2013.37)	0.674
Maximal stress (MPa)	19.35 (5.21-35.54)	13.30 (5.21-24.23)	0.046∗
Stiffness (N/mm)	124.12 (50.58-263.88)	111.57 (34.61-279.56)	0.173

## 4 Discussion

This study demonstrated superior biomechanical outcomes in the repair group utilizing the PTSBR technique, specifically higher Young's modulus (indicating greater tensile stiffness) and increased maximal stress compared to the non-repair group. These findings suggest that the PTSBR technique offers biomechanical advantages that could potentially translate into improved functional outcomes in clinical settings. Additionally, the repair group exhibited a reduction in tendon thickness, which may reflect a more compact and streamlined tendon profile due to the partial-thickness incision inherent in the technique.

The concern with leaving the patellar tendon defect unrepaired relates to the potential for altered tendon integrity and load distribution, which may contribute to quadriceps weakness and impaired extensor mechanism function.^19^^,^^20^ In more severe cases, patellar tendon rupture following BPTB ACL reconstruction has also been reported. However, this complication remains uncommon, with large clinical series describing an incidence generally ranging from approximately 0.05% to 0.2%.^20^^,^^21^ Given this low incidence and the absence of clear evidence demonstrating biomechanical or clinical benefit of routine donor-site repair, management of the patellar tendon defect remains heterogeneous in current practice. Many surgeons elect to leave the defect unclosed, whereas others perform simple side-to-side approximation or superficial paratenon closure rather than a structural repair.

While repair is often thought to restore biomechanical strength and prevent issues like patellar tendon rupture, findings in previous studies challenge this assumption. Sobieraj et al. reported no significant differences in load at failure, engineering failure stress, stiffness, or engineering modulus between repaired and unrepaired tendons.^8^ Additionally, clinical studies, including those by Adriani^11^ and Brandsson,^12^ have shown that closing the defect after patellar tendon harvesting does not significantly affect the extensor apparatus. This conclusion is supported by findings from clinical assessments, as well as imaging evaluations using X-rays, ultrasound, and MRI.^14^, ^15^, ^16^^,^^22^ Some reports even suggest that repair may worsen outcomes; Cerullo et al. found that closing the defect can lead to exuberant scar formation that compromises the entire patellar tendon.^13^ Ultimately, a systematic review of RCTs concluded that there are no statistically significant or clinically relevant differences between repaired and unrepaired patellar tendon defects after ACLR with BPTB autografts, although the methodological quality of these studies was limited.^17^

Adam et al. reported that in the conventional non-repair technique, removal of the BPTB autograft led to irreversible shortening of the remaining two-thirds of the patellar tendon within the first 12 postoperative weeks. Additionally, histological studies have demonstrated that leaving the tendon defect unrepaired can result in extensive fibrosis in the infrapatellar fat pad, significantly compromising the mechanical properties compared to the original tendon.^10^^,^^23^ To address this, we designed the partial-thickness tenotomy and tendon defect closure to induce tendon healing in the midline area, rather than allowing healing through fibrotic tissue formation.

**Fig. 4:**
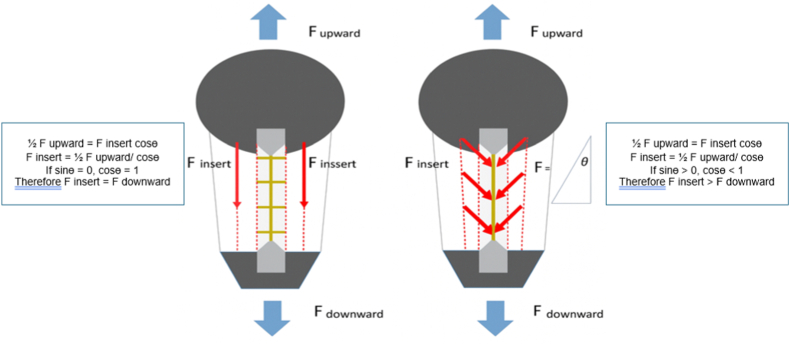
Force Distribution and Mechanical Advantage in Repaired and Non-Repaired Tendons The diagram illustrates the force distribution in patellar tendons under loading conditions. The left panel depicts the force direction in the non-repaired group, where the insertion force (F_insert) is equal to the upward force (F_upward) multiplied by the cosine of the angle (cos θ). When sin θ = 0, cos θ = 1, resulting in minimal load-sharing. The right panel demonstrates the force distribution in the repair group using the Partial Thickness Split and Bridging Repair (PTSBR) technique. Here, the longitudinal alignment of tendon fibers allows for improved load-sharing across the repair site.

The novelty of PTSBR lies in its integration of biomechanical principles of fiber orientation and load-sharing. By introducing a controlled partial-thickness incision and bridging the superficial tendon fibers across the central defect, the technique redirects tensile forces along a more favorable line of pull. This “force-line optimization” reduces stress concentration at the defect margin and distributes load across a broader tendon surface. The use of high-strength Ethibond sutures tied with reinforced knots further enhances fixation security. These biomechanical principles explain the observed improvements in Young's modulus and maximal stress in the repair group. Moreover, based on trigonometric principles, the change in force direction observed in the repair group provided a mechanical advantage, resulting in greater immediate strength of the tendon fibers (Fig. 4). By ensuring better alignment of the repaired tendon fibers and promoting improved load-sharing, the PTSBR technique directly addresses the limitations of conventional edge-to-edge repairs. If validated in future clinical studies, this approach could lead to superior functional outcomes and supports re-evaluating the role of patellar tendon repair in ACLR procedures.

The absence of a significant difference in patellar tendon length between the repair and non-repair groups suggests that the PTSBR technique does not induce acute tendon shortening that could predispose to postoperative patella baja. This distinction is important, as excessively tensioned donor-site closure has been theorized to alter patellar height and potentially affect patellofemoral biomechanics.^18^^,^^23^^,^^24^ The PTSBR configuration was designed to enhance fiber alignment and load-sharing without creating rigid approximation or excessive constraint. Although alterations in patellofemoral mechanics remain a theoretical concern with any donor-site repair, the present findings indicate that this technique does not acutely compromise tendon length. The long-term biomechanical and clinical implications, however, require further investigation.

This study has several limitations. First, a direct side-to-side repair control group was not included. Although prior randomized and observational studies have demonstrated no clear clinical advantage of conventional donor-site closure compared with non-repair,^12^^,^^17^ the absence of a direct comparative arm prevents definitive conclusions regarding the relative superiority of the PTSBR technique over traditional edge-to-edge suturing. Future biomechanical and clinical investigations incorporating a three-arm design would provide a more comprehensive comparison.

Second, patellofemoral contact pressure and dynamic joint kinematics were not directly measured. While previous cadaveric studies have reported no significant differences in patellofemoral contact pressure between repaired and unrepaired tendons following central-third harvest,^9^ the distinct structural configuration of the PTSBR construct may influence force transmission differently. Dedicated biomechanical studies using contact pressure mapping and dynamic tracking analysis are warranted to further clarify its effects on patellofemoral mechanics.

Third, this study evaluated only immediate time-zero biomechanical properties under controlled laboratory conditions. The biological processes of tendon healing, remodeling, scar formation, and long-term adaptation were not assessed.^10^^,^^23^ These factors may substantially influence the mechanical behavior and functional performance of the tendon over time.

Finally, the sample size was limited to six cadavers (12 knees), although it was adequately powered based on prior data. Additionally, the specimens were elderly, which may limit generalizability to the younger and more active population typically undergoing ACL reconstruction.

In conclusion, the PTSBR technique demonstrated improved biomechanical properties compared with non-repair, including greater tensile stiffness and maximal stress under controlled laboratory conditions. By incorporating partial-thickness splitting and bridging of the superficial tendon layers, this configuration may enhance load-sharing across the patellar tendon defect without inducing acute shortening. These findings provide biomechanical support for further investigation of structurally modified donor-site repair strategies following BPTB graft harvest.

## Consent to participate

Not applicable. Cadaveric specimens were obtained through an institutional body donation program, for which informed consent had been provided by donors or their next of kin in accordance with institutional regulations.

## Availability of data and materials

The datasets generated and/or analyzed during the current study are available from the corresponding author on reasonable request.

## Ethics approval

This cadaveric biomechanical study was approved by the Institutional Review Board (Protocol Number: ID 06-59-56).

## Consent for publication

Not applicable.

All authors have read and approved the final manuscript and agree to be accountable for their respective roles.

## Ethics approval

This study was approved by the Committee on Human Rights Related to Research Involving Human Subjects, Faculty of Medicine Ramathibodi Hospital, Mahidol University (Approval No. MURA2016/415), and was conducted in accordance with the Declaration of Helsinki.

## Funding

This study was supported by a research grant from the Faculty of Medicine Ramathibodi Hospital, Mahidol University (Grant No. ID 06–59–56).

## Conflict of interest

The authors declare that they have no competing interests related to the content of this article.
